# What Can We Obtain from Mental Health Care? The Dynamics of Physical and Mental Health

**DOI:** 10.3390/ijerph16173098

**Published:** 2019-08-26

**Authors:** Sung-Joo Yoon

**Affiliations:** Korea Institute of Public Finance, 336, Sicheong-daero, Sejong-si 30147, Korea; sjyoon@kipf.re.kr; Tel.: +82-44-414-2220

**Keywords:** mental health, physical health, dynamic interaction, health expenditures, conditionally correlated random effects model

## Abstract

This study analyzes the dynamic interaction of an individual’s physical and mental health using the German Socio-Economic Panel and the Cross-National Equivalent File of Germany. Its main objective is to find a way to reduce people’s health expenditure by examining the magnitude of the interdependence between physical and mental health. For the analysis, this study develops a dynamic correlated random effects model. We create two aggregate health measures (aggregate physical health and aggregate mental health) with four submeasures each, which provides a better understanding of changes in an individual’s health status by capturing additional information that cannot be analyzed at the aggregate level. There is clear evidence that the persistence of a mental health condition is less than that of a physical health condition. Moreover, the impact of previous mental health on current physical health is greater than that of previous physical health on current mental health. This suggests that individuals can reduce their expenditures on physical health problems by focusing on the treatment of mental problems when they first arise. Finally, the Government’s attention and support toward mental health care would lead to a reduction in health expenditures and eventually improve the sustainability of the nation’s health system.

## 1. Introduction

Mental health is regarded as an integral and essential component of health, and the dimension of mental health is explicitly incorporated in the World Health Organization (WHO) definition of health, as evident from its constitution: “Health is a state of complete physical, mental and social well-being and not merely the absence of disease or infirmity.”

This definition suggests that mental health is more than the absence of mental disorders or disabilities, and that people should not take mental problems such as depression, loneliness, anxiety, and stress—that occur frequently—lightly.

Some mental health problems are known as a risk factor for suicide, substance use, and several conditions such as stroke and coronary heart disease. Mental health problems are not restricted to personal health and well-being alone. They are highly related to social and economic issues as well. Mental health is closely connected to substance use and alcohol consumption, which not only affect mental and physical health, but also financial status. Furthermore, poor mental health may negatively affect relations with family or friends, and may sometimes lead to social crimes.

We also cannot expect a high level of workplace productivity from individuals suffering from mental health problems. According to WHO [1], in many developed countries, mental health problems cause 35–45% of workplace absenteeism. Considering that family members are usually the primary caregivers for people with mental health problems, mental health is associated with the quality of life as well as reduced household income of those families with people who suffer from mental health problems. The members of society mainly finance the cost of treatment via taxation or insurance, reducing the productivity of society, which will ultimately negatively affect the national and world economies.

Given the importance of mental health problems, health economists and medical researchers have become interested in people’s mental health status along with their physical health status; however, studies on mental health are still in their infancy. Moreover, whereas several studies examine the dynamics of general health, few consider the dynamic interaction between physical and mental health. Although medical doctors and health experts claim, based on patient data, that both measurements are related to each other, few studies have covered the general population.

The objective of this study is to find a way to reduce health expenditures by analyzing the individual lagged effects and cross effects of physical and mental health under a dynamic system. We first examine the lagged effects of both physical and mental health, that is, the effect of previous physical and mental health on current physical and mental health, each. Then we examine the cross-effects of previous mental health on current physical health status and vice versa. The magnitude of the state dependence would indicate how the mechanism of people’s health status changes, from which we could obtain some meaningful implications for reducing health care expenditures. We also consider several well-being and socio-economic variables as control variables, as many studies have shown the relationship between these variables and health status.

The results of this study clearly show that the persistence of mental health conditions is less than that of physical health conditions. Moreover, the magnitude of the impact of previous mental health on current physical health is larger than that of previous physical health on current mental health. This suggests that individuals can reduce their health care expenditures toward physical health problems by focusing on the treatment of mental health problems at their onset, which would improve sustainability in the nation’s health system as well.

For empirical modeling, this study relies on a conditionally correlated random effects (CCRE) model with dynamic specification, allowing us to account for the initial conditions. It uses subsamples of the German Socio-Economic Panel (GSOEP) 95% 2008 version and the Cross-National Equivalent File (CNEF) of Germany, as Germany provides good access to mental-health-related resources and services, and thus, we expect that it would reduce the possible bias that arises when people fail to recognize their mental health problems. Health measures in this study are based on the measures included in the Short-Form Health Survey Measurement model, version 2 (SF-36v2). The SF-36 provides two aggregate summary measures of physical and mental health status, and each has four subcomponent measures. Therefore, unlike previous studies, this study not only shows how two aggregate levels of health status are influenced by various variables, but also explains how each aggregate level is affected via its lower-level measures. This could provide a deeper understanding of the mechanism of changes in health status.

## 2. Literature Review

Several studies exist on the association between physical and mental health, many of which are led by medical researchers. For example, both Osborn [2] and Phelan et al. [3] consider the physical health of persons with mental diseases. Osborn [2] examines the physical health of psychiatric patients and finds that patients experience increased mortality, and mortality associated with diverse physical conditions. Phelan et al. [3] attempt to explain the higher mortality of persons with mental diseases and a high rate of substance misuse; however, they do not provide clear evidence, as they fail to compare patients with people from similar social backgrounds.

Some studies examine the effect of physical fitness or activity on mental health. Folkins and Sime [4] suggest that physical training leads to improved mood, self-concept, and work behavior, and Paluska and Schwenk [5] find that increased physical aerobic exercise or strength training significantly reduces depressive symptoms.

Recently, researchers have begun to treat physical and mental health as separate health measures and have also compared these measures. Using the Health and Retirement Study (HRS) data, Dhaval et al. [6] analyze the influence of retirement on individuals’ physical and mental health status, and show that retirement causes more problems in terms of physical health rather than mental health. However, Wray [7] uses the same data as Dhaval et al. [6] and asserts that mental health status is an important predictor of labor force exit rates for older workers.

Many psychologists and economists have studied the relationship between job and health; some of the latest studies have been undertaken with new methods and approaches [8,9,10,11,12]. For example, Fischer and Sousa-Poza [9] evaluate the association between job satisfaction and workers’ health status using a fixed effects model, controlling for unobserved individual heterogeneity with panel data.

Besides job satisfaction, the relationship between income level and health status has also been of interest to researchers; however, the direction of causality remains an open issue. On one hand, poor health is regarded as a factor that has a negative impact on income poverty, as poor health reduces the amount of labor supply and wage earnings [13,14,15,16]. On the other hand, low income could cause poor health because of malnutrition and the difficulty of accessing medical resources or services [17].

Further, several health-related studies have used dynamics and the correlated random effects model. Contoyannis et al. [18] investigated the dynamics of individuals’ general health status with the individual-specific effects and find that state dependence dramatically reduces the impact of individual heterogeneity. Halliday [19] analyzes the evolution of health status over a life cycle and finds that individual characteristics can have a far-reaching impact on health status by allowing for unobserved heterogeneity and state dependence.

Ohrnberger et al. [20] and Ohrnberger et al. [21] considered the relationship between mental and physical health in a dynamic specification similar to this study. Ohrnberger et al. [20] investigated the dynamic interaction between mental and physical health using a sample of the elderly population in six waves of the English Longitudinal Study of Ageing (ELSA, 2002–2013). For the analysis, they used conditional linear and nonlinear random-effects regression models, and found both mental and physical health outcomes are moderately state-dependent; better previous metal health improves current physical health significantly; better previous physical health has a larger effect on current mental health. Ohrnberger et al. [21] employed mediation analysis methods using data similar to Ohrnberger et al. [20]. Here, they showed that past mental (physical) health has a significant direct and indirect impact on current physical (mental) health, where direct effect means lagged effect and indirect effect means lifestyle choices and social capital. However, in both studies, the subjects are restricted to old people; furthermore, the index of mental (Centre for Epidemiological Studies Depression: CES, 0–8 scale) and physical (Activities of Daily Living: ADL, 0–6 scale) health scales are different, so it is not easy to compare the magnitudes of mental and physical health effects.

## 3. Data and Methods

### 3.1. Data

For the empirical analysis, we use the German Socio-Economic Panel (GSOEP) data. A possible problem in a mental health study is that unlike physical health problems, people may not recognize their mental health problems, or they may place less importance on their mental problems. This problem would occur with higher probability in lower income countries, as it is less likely that they allocate enough health resources to mental health services, policies, or programs. According to the World Bank’s criteria, Germany belongs to a high-income group country, and the people there have better access to mental health services and information. Therefore, we expect that this study would reduce the possible bias that arises when individuals fail to recognize their mental health problems, by using the German dataset.

Furthermore, in health-related studies, the choice of health measures is important for keeping an objective viewpoint. The measures in this study are based on the Short-Form Health Survey Measurement model (SF-36), which is a generic measure whose usefulness in surveys of general and specific population groups has been established, and GSOEP’s questionnaire includes many parts of the SF-36 criteria. Note that the GSOEP employed in this study uses a randomly chosen 95% of observations in the original dataset, referred to as the GSOEP 95% 2008 version. Additionally, we incorporate four variables that are not in the GSOEP (having trouble in dressing, getting out of bed, shopping, doing housework) into our dataset from the Cross-National Equivalent File (CNEF) of Germany. Many health-related variables have been collected biennially, so the merged dataset is composed of biennial data from 2002 to 2008.

For proxy variables related to health status, we create eight component measures: Physical Functioning (PF), Role-Physical (RP), Bodily Pain (BP), General Health (GH), Vitality (VT), Social Functioning (SF), Role-Emotional (RE), and Mental Health (MH), and two aggregate summary measures: Aggregate Physical Health (APH) and Aggregate Mental Health (AMH), considering the idea and structure of the health measures in the SF-36. The eight recoded submeasures have bivariate distributions in line with the econometric framework of this study. If an individual has no problem as per the related questionnaires, it implies that the individual is in good health condition in relation to the submeasure, and this is indicated as 1. However, if the person has at least one problem, then he/she is regarded as having a problem in the submeasure, and this is indicated as 0. For example, if an individual has no problem concerning climbing stairs, doing tasks, dressing, getting out of bed, shopping, and doing housework, then his/her PF is indicated as 1, implying good physical functioning; if there is at least one problem, then his/her PF is indicated as 0, implying bad physical functioning (Table 1).

**Figure 1:**
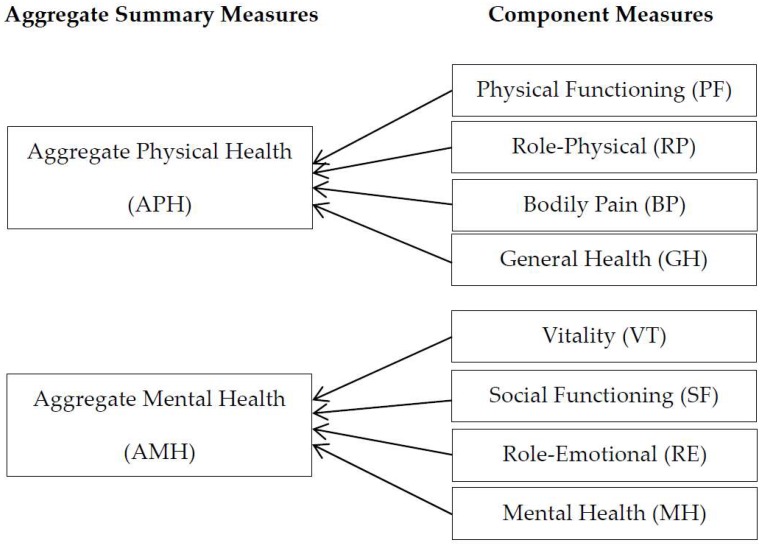
Aggregate health measures and related submeasures.

The two aggregate summary measures, APH and AMH, are also dummy variables; Table 1 shows how they are generated. An individual who has no problem in all physical-related submeasures is considered to be in good physical health condition. In other words, an individual’s APH is indicated as 1 if all PF, RP, BP, and GH are 1. However, if he/she has at least one problem among the four physical-related submeasures, then he/she is regarded to be in poor physical health status, indicated as 0. The other aggregate measure, AMH, is also generated in a similar way (Figure 1).

In this study, we consider four well-being variables: satisfaction with job, satisfaction with housework, satisfaction with household income, and satisfaction with the amount of leisure time as explanatory variables. Originally, each variable is coded on an 11-point scale, from 0 to 10, where 0 implies the lowest level of satisfaction, and 10 as the highest in the GSOEP. We recode it into two levels, high satisfaction and low satisfaction, by recoding 1 if it is six or higher, and 0 otherwise. We also consider several socio-economic variables, namely years of education, white-collar, body mass index, family size, and so on. Both years of education and white-collar reflect an individual’s social status. The white-collar implying a person’s job type is an indicator variable coded as 1 if the individual is a white-collar worker, and 0 otherwise. The body mass index is calculated as weight in kilograms divided by the square of height in meters. Additionally, the empirical model includes variables such as age, marital status (single, divorced, and widowed), and location where individuals live (west, north, and south). Note that, for the time-averaged variables used in the empirical model, we choose the time average of four-satisfaction-related variables, and time averages of age and body mass index.

We exclude individuals who are less than 20 and over 65 years old to reduce age effects. Further, we exclude the self-employed from the dataset, as the satisfaction levels of the self-employed are usually different from those of the employed. Thus, our sample is composed of a total four waves of 15,121 individuals (53% women; 47% men), totaling 47,268 observations (Table 2).

### 3.2. Concept of Relationship between Mental and Physical Health

**Figure 2:**
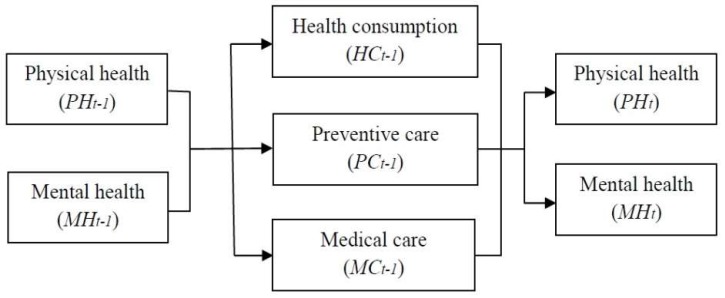
The relationship between previous and current health status

Before explaining the empirical strategy, we need to consider the relationship between mental and physical health (Figure 2).

Given physical health ($PH_{t-1}$) and mental health ($MH_{t-1}$) in the previous period, individuals determine the amount of health-related expenditures, such as health consumption $\left(HC_{t-1}\right)$, preventive care $\left(PC_{t-1}\right)$, and medical care $\left(MC_{t-1}\right)$ considering their budget constraints. The previous consumptions affect current physical health ($PH_{t}$) and mental health ($MH_{t}$), where the health-related expenditure considering previous physical (mental) health affects not only current physical (mental) health (own effects) but also current mental (physical) health (cross effects). For example, it is well known that physical fitness or activity not only enhances physical health status, but also reduce depressive symptoms, and improves mood and self-concept (Folkins and Sime [4]; Paluska and Schwenk [5]).

Furthermore, previous preventive care and medical care tend to have positive effects on current physical and mental health $\left(\frac{\partial PH_{t}}{\partial PC_{t-1}}>0,\;\frac{\partial MH_{t}}{\partial PC_{t-1}}>0,\;\frac{\partial PH_{t}}{\partial MC_{t-1}}>0,\;\frac{\partial MH_{t}}{\partial MC_{t-1}}>0\;\right)$, but previous health consumption can have a negative influence on current health status depending on the nature of the good, that is, healthy $\left(\frac{\partial PH_{t}}{\partial HC_{t-1}}>0,\;\frac{\partial MH_{t}}{\partial HC_{t-1}}>0\right)$ or hazardous $\left(\frac{\partial PH_{t}}{\partial HC_{t-1}}<0,\;\frac{\partial MH_{t}}{\partial HC_{t-1}}<0\right)$. Accordingly, current physical and mental health is the function of both previous physical and mental health, which allows cross-dynamic effects between mental and physical health, as well as own lagged effects. However, it should be noted that for the empirical analysis, we use a reduced form model, and not a structural model; therefore, the individual effects of health consumption, preventive care, medical care is not identified in this study.

### 3.3. Model with State Dependence

To capture the magnitude of state dependence, we use a dynamic panel-ordered probit model with latent variable specification. We use this specification because we are interested in the person’s underlying health status, which is both unobservable and continuous. The dynamic model we estimate is as follows:

(1) $$y_{h,i,t}^{*}=\rho_{h}y_{h,i,t-1}+\gamma_{h}y_{-h,i,t-1}+x_{i,t}^{\prime }\beta_{h}+\varepsilon_{h,i,t},$$

where $y_{h}$ and $y_{-h}$ represent the physical and mental health status measures, respectively; subscript i represents individuals (i = 1, 2, …, N); t is the time period (t = 1, 2, …, T); and vector x includes well-being and socio-economic variables. Finally, shock ε is composed of two components: individual-specific effects, $\alpha_{h,i}$, and a pure random part, $u_{h,i,t}$.

Generally, we can consider two models for panel analysis: fixed effects and random effects. The random effects model assumes that the correlation between individual-specific effects and covariates is zero, which is quite a strong assumption. Although the fixed effects model allows a possible correlation, it does not provide the estimates of time-invariant variables. Considering the short panel period, the effect of initial values, which are time invariant, would be considerable in this study. Therefore, we use a conditionally correlated random effects (CCRE) model, allowing for the effect of both possible correlation and initial values.

In the CCRE model, individual-specific effect (α) can be decomposed into three parts: initial conditions ($y_{0}$), correlated heterogeneity (z), and a pure random part (η), as follows:

(2) $$\alpha_{h,i}=\alpha_{h,0}+\alpha_{h,1}y_{h,i,0}+z_{i}^{\prime }\alpha_{h,2}+\eta_{h,i},$$

where $\eta_{h,i}|\;\left(y_{h,i,0},z_{i}\right)\sim$Normal ($0,\sigma_{h,\eta }^{2}$) over i, and it could be interpreted as unobserved heterogeneity uncorrelated with the regressors [22]. An individual’s initial health condition would be considered as a predictive value in a health study, especially in a short panel. Moreover, the model can capture some aspects of individual-specific microeconometric heterogeneity by incorporating the initial value into the unobserved heterogeneity. Further, if we can find some suitable sufficient statistics for the covariates that can allow for the possible correlation between individual-specific effects and covariates, then the remaining unobserved heterogeneity can be treated as a purely random part [22]. For the sufficient statistics, we create time-averaged values (z) of several time-variant variables ($x_{c}$) in vector (x) in order to correlate several covariates and individual heterogeneity [23], that is, $z_{i}=\frac{1}{T}\sum_{t=1}^{T}x_{c,i,t}.$

Unlike the fixed effects model or conditional maximum likelihood estimation, which are alternatives to the CCRE model, the applications of the CCRE model have been restricted to balanced panel data. However, Wooldridge [24] shows that the CCRE model can also be used for an unbalanced panel in the case of an ordered probit model, and suggests that although it is not necessary, including time averages of key variables in the model helps to estimate more precise key parameters.

It is challenging to estimate the magnitude of true state dependence when there exists unobserved heterogeneity because we need to condition on unobserved heterogeneity for calculating the correlation between dependent and lagged dependent variables. However, in the CCRE model, we can identify some parts of the individual-specific effects, which would partially reduce the amount of spurious state dependence in the estimation by conditioning the identified parts of individual-specific effects, along with other exogenous variables (for details, see reference [25]). Therefore, we obtain the following dynamic CCRE model including the initial value by substituting Equation (2) into Equation (1) as follows:

(3) $$y_{h,i,t}^{*}=\rho_{h}y_{h,i,t-1}+\gamma_{h}y_{-h,i,t-1}+x_{i,t}^{\prime }\beta_{h}+\alpha_{h,0}+\alpha_{h,1}y_{h,i,0}+z_{i}^{\prime }\alpha_{h,2}+\eta_{h,i}+u_{h,i,t},$$

where $\eta_{h,i}|y_{h,i,0}$, $z_{i}\sim \mathrm{Normal}\left(0,\sigma_{h,\eta }^{2}\right)$ over i, and $u_{h,i,t}|(y_{-h,i,t-1},x_{i,t},{\;y}_{h,i,t-1},\;y_{h,i,t-2},$…,$y_{h,i,0,\;}\;\eta_{h,i})\sim \mathrm{Noraml}$ $(0,\sigma_{h,u}^{2}$) over both i and t.

In the model, we assume that both physical and mental health status have two levels: good status is indicated as 1 and poor status as 0. Note that we cannot observe the latent outcome, $y_{h,i,t}^{*}$, but we can observe the category indicator

(4) $$y_{h,i,t}=1\left\{y_{h,i,t}^{*}\geq 0\right\}.$$

Then, the model can be specified in two ways depending on the response variable:

(5) $$\begin{array}{c} y_{p,i,t}=1\{y_{p,i,t}^{*}=\rho_{p}y_{p,i,t-1}+\gamma_{p}y_{m,i,t-1}+x_{i,t}^{\prime }\beta_{p}\\ +\alpha_{p,0}+\alpha_{p,1}y_{p,i,0}+z_{i}^{\prime }\alpha_{p,2}+\eta_{p,i}+u_{p,i,t}\geq 0\}, \end{array}$$

and

(6) $$\begin{array}{c} y_{m,i,t}=1\{y_{m,i,t}^{*}=\rho_{m}y_{m,i,t-1}+\gamma_{m}y_{p,i,t-1}+x_{i,t}^{\prime }\beta_{m}\\ +\alpha_{m,0}+\alpha_{m,1}y_{m,i,0}+z_{i}^{\prime }\alpha_{m,2}+\eta_{m,i}+u_{m,i,t}\geq 0\}, \end{array}$$

where subscript p and m represent physical health and mental health, respectively. Furthermore, the model has a dynamic probit specification, and the density of ($y_{h,i,1},y_{h,i,2},\ldots ,y_{h,i,T})$ given the values of $y_{-h,i,t-1},x_{i,t},y_{h,i,0}$, and $\eta_{h,i}$ is

(7) $$\prod_{t=1}^{T}\left\{\begin{array}{c} \Phi {\left(\rho_{h}y_{h,i,t-1}+\gamma_{h}y_{-h,i,t-1}+x_{i,t}^{\prime }\beta_{h}+\alpha_{h,0}+\alpha_{h,1}y_{h,i,0}+z_{i}\alpha_{h,2}+\eta_{h,i}\right)}^{y_{h,i,t}}\times \\ {\left[1-\Phi \left(\rho_{h}y_{h,i,t-1}+\gamma_{h}y_{-h,i,t-1}+x_{i,t}^{\prime }\beta_{h}+\alpha_{h,0}+\alpha_{h,1}y_{h,i,0}+z_{i}\alpha_{h,2}+\eta_{h,i}\right)\right]}^{1-y_{h,i,t}} \end{array}\right\}.$$

Next, integrating out $\eta_{h,i}$ in Equation (7) gives the density of ($y_{h,i,1},y_{h,i,2},\ldots ,y_{h,i,T})$ given the values $y_{-h,i,t-1},x_{i,t},$ and $y_{h,i,0}$ as follows:

(8) $$\int_{-\infty }^{+\infty }\left(\overset{T}{\underset{t=1}{\prod }}\left\{\begin{array}{c} \Phi {\left(\begin{array}{c} \rho_{h}y_{h,i,t-1}+\gamma_{h}y_{-h,i,t-1}+x_{i,t}^{\prime }\beta_{h}+\\ \alpha_{h,0}+\alpha_{h,1}y_{h,i,0}+z_{i}^{\prime }\alpha_{h,2}+\eta_{h,i} \end{array}\right)}^{y_{h,i,t}}\times \\ {\left[1-\Phi \left(\begin{array}{c} \rho_{h}y_{h,i,t-1}+\gamma_{h}y_{-h,i,t-1}+x_{i,t}^{\prime }\beta_{h}+\\ \alpha_{h,0}+\alpha_{h,1}y_{h,i,0}+z_{i}^{\prime }\alpha_{h,2}+\eta_{h,i} \end{array}\right)\right]}^{1-y_{h,i,t}} \end{array}\right\}\right)\times \left(\frac{1}{\sigma_{\eta_{h}}}\right)\phi \left(\frac{\eta_{h}}{\sigma_{\eta_{h}}}\right){d\eta }_{h},$$

where Ф(·) and ф(·) represent the standard normal cumulative distribution function and standard normal distribution function, respectively. Finally, we can obtain estimates by maximizing the likelihood function using the Newton–Raphson method (for details, see reference [26]).

Note that there are some limitations to our model. First, the model does not allow for serial correlation in the idiosyncratic error terms, although serial correlation is known as a contributor to the magnitude of state dependence. However, the results of pooled estimation that capture the serial correlation, while it ignores the variation of unobserved heterogeneity, do not change our conclusion. It suggests that serial correlation may not be an important issue in this study. Another limitation concerns individual heterogeneity; individual heterogeneity in Equations (5) and (6) are related to each other due to time-average components; however, the model does not allow for the correlation between $\eta_{p,i}$ and $\eta_{m,i}$, which are treated as purely random parts in the individual-specific component. Considering that the individual-specific effect also affects the size of state dependence, no relation between the two pure parts might distort the magnitude of true state dependence. However, note that the common sufficient statistics in the individual-specific effect not only connect two equations, but also decrease the amount of a pure random part; thus, we may expect that the impact of the pure random part on the estimation may not be considerable.

### 3.4. Estimating Average Partial Effects

We calculate the average partial effects to estimate the magnitude of state dependence and some other covariates. For example, to obtain the magnitude of state dependence of physical health state (h), we estimate the probability of being in a good physical health state in the current period when the individual was or was not in a good physical health state in the previous period. Then, the average partial effect of $y_{h,i,t-1}$ on $y_{h,i,t}$ is defined as

(9) $$\mathrm{APE}=E\left\{P\left(y_{h,i,t}^{\left(1\right)}|y_{h,i,t-1}^{\left(1\right)},y_{-h,i,t-1},x_{i,t},y_{h,i,0}\right)-P\left(y_{h,i,t}^{\left(1\right)}|y_{h,i,t-1}^{\left(0\right)},y_{-h,i,t-1},x_{i,t},y_{h,i,0}\right)\right\},$$

where the expectation is with respect to all characteristics over i, and subscript −h indicates the mental health state.

Thus, the average partial effects are consistently estimated by

(10) $$\hat{\mathrm{APE}}=\frac{1}{N}\overset{N}{\underset{i=1}{\sum }}\{\begin{array}{c} \Phi \left({\;\hat{\rho }}_{h}y_{h,i,t-1}^{\left(1\right)}+{\;\hat{\gamma }}_{h}y_{-h,i,t-1}+x_{i.t}^{\prime }{\hat{\beta }}_{h}+{\;\hat{\alpha }}_{h,0}+{\;\hat{\alpha }}_{h,1}y_{h,i,0}+z_{i}^{\prime }{\hat{\alpha }}_{h,2}\right)\\ -\Phi \left({\;\hat{\rho }}_{h}y_{h,i,t-1}^{\left(0\right)}+{\;\hat{\gamma }}_{h}y_{-h,i,t-1}+x_{i.t}^{\prime }{\hat{\beta }}_{h}+{\;\hat{\alpha }}_{h,0}+{\;\hat{\alpha }}_{h,1}y_{h,i,0}+z_{i}^{\prime }{\hat{\alpha }}_{h,2}\right) \end{array}\}.$$

## 4. Results and Discussion

### 4.1. State Dependence

We need to consider how the previous health state affects the current health state before analyzing the magnitude of state dependence. Obviously, previous intervention affects previous health status, which affects current health status. Therefore, the previous shock plays an important role here. When there are interventions in the previous period, they affect previous physical and mental health, which ultimately affect current physical and mental health. Similarly, previous mental health also influences the current physical and mental health statuses. Therefore, we can say that current health states are influenced by previous interventions in the dynamic system via previous health states. We may not make inferences about the causality in the analysis of well-being and socio-economic effects, as the econometric model is a dynamic reduced form. However, we can infer the causal effect in the physical and mental health interaction because we consider the relationship between previous and current health status, so the causal direction is evident.

Table 3 shows the positive and significant effects of lagged dependent variables for both APH and AMH. Individuals who were in good physical health condition in the previous period have a probability of being in good physical health in the current period, that is, about 12.4 and 11.7 percentage points higher for men and women, respectively, than those of individuals who were in poor health condition in the previous period. In the case of mental health, they are about 8.4 and 8.7 percentage points higher for men and women, respectively. This implies that the magnitudes of the contribution of lagged physical health on current physical health state are larger than those of lagged mental health on current mental health state. This may occur if physical health problems are more persistent than mental health problems. In other words, recuperation from physical health problems takes relatively longer; however, mental health problems can be overcome relatively quickly compared to physical health problems.

Moreover, for all eight submeasures, state dependence is evident, as PF and VT show the largest state dependence in the physical- and mental-related submeasures (Table 4, Table 5, Table 6 and Table 7), respectively; the gap is about 18.1 (men) and 13.2 (women) percentage points in terms of PF (Table 5), and about 7.7 (men) and 8.7 (women) percentage points in terms of VT (Table 7). This implies that previous physical functioning and vitality considerably affect current physical and mental health, respectively.

The cross effects between APH and AMH are both positive and significant (Table 3). This implies that there is no unilateral direction of causality; current physical and mental health states are affected by previous mental and physical health states, respectively. Having been in good physical health state in the previous period increases the probability of being in good mental health state in the current period by 5.7 and 6.8 percentage points for men and women, respectively. Further, the increased probability of being in good physical health state in the current period are 12.2 and 9.0 for men and women, respectively, when an individual was in good mental health state in the previous period. Although the magnitudes of the effects are overall lesser than those of lagged effects, the sizes are still not ignorable, considering the sizes of own lagged effects. This suggests that individuals can improve physical health status by managing their mental health status, and vice versa.

More importantly, the effect of previous mental health on current physical health status is larger than that of previous physical health on current mental health. In other words, physical health is largely influenced by mental health, but the effect of physical health on mental health is relatively low. The cross effect of mental health on physical health is quite large for men, which means that men’s physical health state is highly dependent on their mental health. Considering that most health expenditures relate to physical treatment, individuals can reduce their health expenditures by controlling their mental health status.

For the eight submeasures, the cross effect of state dependence is clear and positive (Table 4 and Table 6). Furthermore, the overall partial effects on physical health are larger for men, while the partial effects on mental health are larger for women, which also apply to the aggregate summary levels.

### 4.2. Well-Being

Job satisfaction is statistically significant and positive for both APH and AMH, which is consistent with previous studies that job satisfaction positively affects people’s health status (Table 3). The partial effects of APH are larger than those of AMH for both men and women. For both APH and AMH, the partial effects are larger for men compared to women, and the difference is larger for APH. This implies that individuals’ physical health is more sensitive to their job satisfaction, and the effects are larger for men than for women.

Most physical- and mental-related submeasures are statistically significant (Table 4 and Table 6), and the partial effects of GH and RE are the largest in the physical- and mental-related submeasures, respectively (Table 5 and Table 7). Further, the partial effects of submeasures show something that the aggregate measures fail to show. Although the partial effect of APH is larger than that of AMH, and the partial effect of RE is larger than that of RP, both are submeasures of AMH and APH, respectively. This means that when considering work-related measures, mental health (RE) is more sensitive to job satisfaction than physical health (RP). In addition, Table 7 presents that for mental-related submeasures (VT, SF, and MH), women’s partial effects are larger than that of men. This effect is the opposite of the result from the aggregate levels, which show that the effects are larger for men for both APH and AMH. Therefore, we may say that job satisfaction influences both men’s and women’s physical and mental health; moreover, men’s physical health and women’s mental health are relatively more sensitive to the level of their job satisfaction.

Next, the effect of housework on health status, unlike other satisfaction categories, is not clear; only the effect on men’s physical health is statistically significant at the aggregate level. It also happens in the physical-related submeasures. For satisfaction with household income, only APH is statistically significant at the aggregate level. However, the effects on women’s measures are statistically significant for the entire mental-related submeasures (VT, SF, RE, and MH) and the magnitudes of partial effects are also larger than those of men. As for the satisfaction with leisure time, the effects are positive and statistically significant on both women’s physical and mental health, and the magnitude of partial effect is slightly larger in the case of mental health.

### 4.3. Socio-Economic Factors

The effect of age on APH is statistically significant and negative for both men and women, and its marginal effect is slightly larger for women. Specifically, the effects mainly depend on BP and GH. However, age effects on AMH and its four submeasures—VT, SF, RE, and MH—are not statistically significant. This implies that although an individual’s physical health becomes worse as time passes, his/her mental health does not depend on age. It might be because of learning; people learn from experience to control their mental problems such as stress.

Overall, the effects of education on both APH and AMH are statistically significant, but the direction of both effects is different. The marginal effect of education on APH is positive, which may be interpreted that individuals who received more education can manage their physical health status, which is in line with many previous studies that highly educated people have better health status compared to less educated people. In addition, the effect of BP is clearer than others, and the marginal effect of BP is also larger than other physical-related submeasures. However, the marginal effect on mental health is negative. Specifically, the effects on VT and RE are clear among mental-related submeasures, especially for men. This could be because more educated people face more complex tasks and face larger workloads in cramped offices and relative isolation. These considerations may be associated with stress, depression, and loneliness, thus affecting negatively on mental health.

The white-collar variable shows a similar result to those of years of education. Overall, the results in Table 3 indicate that white-collar workers have better physical health status than industrial workers; however, industrial workers have on average better status than white-collar workers in terms of mental health. The explanations for these results are similar to those for education. The body mass index (BMI) has a negative effect on APH, but the effect on AMH is not clear. Further, the marginal effect on women’s APH is larger than that on men. For all four physical-related submeasures, the effects are negative and statistically significant. In addition, the negative marginal effect is the largest for PF for both men and women. This implies that an overweight or obese condition may first affect physical movement, and then lead to many kinds of obesity-related illnesses such as diabetes, hyperglycemia, hyperlipidemia, and heart disease. These illnesses may negatively affect mental health in the end. The number of persons in a family is associated with women’s health. It has a statistically significant effect on both women’s APH and AMH; however, the direction is opposite. Large family size has a positive marginal effect on women’s APH, including most of its submeasures, but has a negative effect on their AMH.

## 5. Robustness Checks

Here, we investigate the previous analysis with a small subsample, which is divided into two age groups: a younger group consisting of people who are between 20 and 50 years old in 2008, and an older group in which individuals’ ages are between 51 and 65 in 2008. The younger group comprises 9174 individuals (4250 men, 4924 women), and the mean age is 35.31; the older group comprises 5947 people (2744 men, 3148 women), and the average age is 55.73.

Overall, the results (Table 8 and Table 9) are similar to those discussed in Section 4, but many variables lose their statistical significance because of fewer observations. As in the results from the previous sample, most lagged effects in the small sample are clear, and the results are mostly similar to the previous results. A new finding in the small sample is that the physical and mental health of older men is highly correlated. The effect of older men’s mental health on their physical health is large and the adverse effect is also considerable. The average partial effect of previous mental health on current physical health is about 0.13, and that of previous physical health on current mental health is about 0.08. This implies that physical problems in older men are severe enough to negatively affect their mental health; moreover, although they may be good at managing their mental condition, once they face mental problems, then its effect is highly connected to their physical problems.

Furthermore, the average partial effect of mental health on physical health is about 0.11, and that of physical health on mental health is about 0.06 for the younger group. This implies that the effect of mental health on physical health is also considerable for younger people, which suggests that we need to focus on the treatment of mental health to reduce younger people’s possible future health expenditure arising from mental health problems.

## 6. Conclusions

We examined the dynamic interaction of an individual’s physical and mental health, and considered two aggregate health measures (physical health and mental health) with four submeasures each. For the empirical analysis, we used the German Socio-Economic Panel and the Cross-National Equivalent File of Germany, and employed dynamic correlated random effects model to reduce some aspect of individual-specific microeconometric heterogeneity. This is a meaningful contribution in that unlike previous studies, this study not only shows how two aggregate levels of health status are influenced by various variables, but also explains how each aggregate level is affected via its lower-level measures, which could provide a better understanding of changes in an individual’s health status.

We find that there are lagged effects for both physical and mental health; furthermore, there are cross effects between physical health and mental health in the dynamic specification. This means that current physical (mental) health is not only affected by previous physical (mental) health but also previous mental (physical) health status, which suggests that individuals could improve physical (mental) health by managing their mental (physical) health care.

More importantly, we recognize that the magnitude of the physical lagged effect is larger than that of the mental lagged effect, and the magnitude of the mental cross effect is larger than that of the physical cross effect. This means that the magnitude of the impact of previous physical health on current physical heath is larger than that of previous mental health on current mental health; the magnitude of the impact of previous mental health on current physical health is larger than that of previous physical health on current mental health. Overall, we find these results for both the aggregate level and the submeasures. Generally, most health expenditures relate to physical treatment, and many people fail to recognize their mental problems or pay less attention to curing their mental health problems compared with the care given to physical health problems. Therefore, the empirical results imply that the significant expenditures to treat physical health problems could be reduced by paying greater attention to mental health treatment. The empirical results also show that, overall, the cross effects of previous mental health on current physical health is quite large for men, while the cross effects of previous physical health on current mental health is relatively larger for women. This implies that mental care could contribute to a great improvement of men’s physical health, while physical care could contribute to a large enhancement of women’s mental health.

The implication is not restricted to the individual level. It is essential to promote the importance of mental health at the national level, so people recognize their mental problems and actively participate in the treatment of mental problems at an early stage. Furthermore, as previous mental health has a greater impact on the current physical health of younger people, mental health problems should be addressed seriously. Considering this, the government’s attention and support toward people’s mental health would ultimately reduce national health expenditures and contribute to maintaining the sustainability of the nation’s health care system.

We used well-being variables as control variables along with health variables in the empirical model. In order to conduct an in-depth study, we need to consider many factors that might affect the well-being variables. Even though we did not consider these factors in this study, we could obtain some meaningful results. Especially, we find that job satisfaction is statistically significant and positively affects both physical and mental health. The effects are larger for men compared to women for both physical and mental health status, and the difference is larger for mental health. This implies that individuals’ health could be improved by enhancing their job satisfaction, which could eventually affect national economic growth as individuals’ health affect their productivity; individuals’ productivity affects companies’ competitive advantage; companies’ competitive advantage affects the nation’s productivity and growth. Therefore, companies need to improve their working environments and conditions by managing programs for physical and mental health, relationships between employees, and so on, which could contribute to improving individuals’ job satisfaction. Government should provide guidelines for such programs, and financial and institutional support, such as tax subsidies for company programs that contribute to improving job satisfaction.

This study considers various aspects, but there are several limitations. We examined only short-term effects in this study, but the long-term effects also need to be considered. Next, we implicitly explained the effects of health dynamics on health expenditures and did not explicitly analyze the effects using econometric models. These issues are important, but they cannot be analyzed with our econometrics tool in this study. We leave theses important issues for further study.

## Figures and Tables

**Table 1:** Questionnaire of each component measure.

Aggregate	Component	Questionnaire	Recode
Measures	Measures		
APH	PF	state of health affects ascending stairs	APH = 1{PF = RP = BP = GH = 1}
		state of health affects tiring tasks	
		has trouble dressing	
		has trouble getting out of bed	
		has trouble shopping	
		has trouble doing housework	
	RP	accomplishes less due to physical problems limitations due to physical problems	
	BP	strong physical pain in the past four weeks	
	GH	current health	
APH	VT	pressed for time in the past four weeks	AMH = 1{VT = SF = RE = MH = 1}
		run-down, melancholy in the past four weeks	
	SF	limited socially due to health	
	RE	accomplishes less due to emotional problems	
		less careful due to emotional problems	
	MH	well-balanced in the past four weeks	

Note: APH = aggregate physical health, AMH = aggregate mental health, PF = physical functioning, RP = role-physical, BP = bodily pain, GH = general health, VT = vitality, SF = social functioning, RE = role-emotional, MH = mental health.

**Table 2:** Descriptive statistics.

Variable	Mean	Standard Deviation	Min	Max	Observations
**Health measures**					
APH	0.3694	0.4826	0	1	47,251
PF	0.5485	0.4977	0	1	47,257
RP	0.6836	0.4651	0	1	47,236
BP	0.7127	0.4525	0	1	47,268
GH	0.5630	0.4960	0	1	47,267
AMH	0.1796	0.3839	0	1	47,264
VT	0.2026	0.4019	0	1	47,266
SF	0.8275	0.3778	0	1	47,260
RE	0.7749	0.4177	0	1	47,246
MH	0.8470	0.3600	0	1	47,261
**Control variable**					
age	43.2466	12.2234	20	65	47,268
satisfaction w. job	0.7845	0.4111	0	1	36,463
satisfaction w. housework	0.7146	0.4516	0	1	35,971
satisfaction w. HH income	0.6826	0.4655	0	1	46,763
satisfaction w. leisure time	0.7284	0.4448	0	1	47,125
years of education	12.4398	2.6752	7	18	45,898
white-collar	0.6593	0.4740	0	1	31,361
body mass index	25.5088	4.4400	11.628	67.202	47,186
family size	2.8984	1.2592	1	13	47,268

Note: APH = aggregate physical health, AMH = aggregate mental health, PF = physical functioning, RP = role-physical, BP = bodily pain, GH = general health, VT = vitality, SF = social functioning, RE = role-emotional, MH = mental health.

**Table 3:** Estimation results: aggregate level.

Variables	Coefficient Estimates				Average Partial Effects			
	APH		AMH		APH		AMH	
	Men	Women	Men	Women	Men	Women	Men	Women
physical (t−1)	0.4257 ***	0.4264 ***	0.2878 ***	0.3520 ***	0.1238 ***	0.1168 ***	0.0574 ***	0.0680 ***
mental (t−1)	0.4184 ***	0.3312 ***	0.3990 ***	0.4336 ***	0.1216 ***	0.0899 ***	0.0836 ***	0.086 7 ***
age	−0.0531 ***	−0.0619 ***	−0.0150	−0.0104	−0.0150 ***	−0.0161 ***	−0.0026 **	−0.0017 *
single	−0.0217	0.0567	0.1648 *	0.0943	−0.0061	0.0148 **	0.0300 ***	0.0159 ***
divorced	0.0619	0.2478 ***	−0.1032	0.0489	0.0175 *	0.0661 ***	−0.0173 ***	0.0082 *
widowed	0.1022	0.1555	0.1917	−0.1527	0.0291	0.0413 ***	0.0365 *	−0.0232 ***
west	0.0014	0.1341 *	0.0090	−0.0248	0.0004	0.0352 ***	0.0016	−0.0040
north	0.0417	0.0092	0.1536	0.0493	0.0118	0.0024	0.0283 ***	0.0082 *
south	0.1308 *	0.1604 **	0.0735	−0.0721	0.0371 ***	0.0420 ***	0.0129 ***	−0.0117 ***
satisfaction w. job	0.3290 ***	0.1725 **	0.2388 **	0.2254 **	0.0952 ***	0.0459 ***	0.0466 ***	0.0411 ***
satisfaction w. housework	0.1549 *	0.0859	0.1224	0.0371	0.0443 ***	0.0226 ***	0.0226 ***	0.0062
satisfaction w. HH income	0.2210 **	0.1441 *	0.0822	0.1314	0.0635 ***	0.0382 ***	0.0149 **	0.0229 ***
satisfaction w. leisure	0.0218	0.1985 ***	0.1722 *	0.3021 ***	0.0061	0.0530 ***	0.0326 ***	0.0571 ***
years of education	0.030 3**	0.0340 ***	−0.0262 *	−0.0177	0.0085 ***	0.0088 ***	−0.0046 ***	−0.0029 ***
white-collar	0.1549 **	0.0614	−0.2092 ***	−0.1390 *	0.0443 ***	0.0161 ***	−0.0328 ***	−0.0213 ***
body mass index	−0.0615 **	−0.0789 ***	0.0349	−0.0115	−0.0173 ***	−0.0205 ***	0.0061 ***	−0.0019 *
family size	0.0228	0.0564 **	−0.0566 *	−0.0575 *	0.0064 ***	0.0147 ***	−0.0099 ***	−0.0094 ***
*Initial condition, and correlated random effect (time average)*								
physical (t = 0)	0.7082 ***	0.8339 ***			0.2276 ***	0.2536 ***		
mental (t = 0)			0.5901 ***	0.6381 ***			0.1284 ***	0.1331 ***
mean (age)	0.0285 *	0.0434 ***	0.0306 *	0.0236	0.0080 ***	0.0113 ***	0.0053 ***	0.0039 ***
mean (job)	0.2305 *	0.3179 **	0.4520 **	0.0783	0.0649 ***	0.0826 ***	0.0788 ***	0.0128 *
mean (housework)	−0.0227	0.2029 *	−0.1104	0.2573 *	−0.0064	0.0527 ***	−0.0192 **	0.0422 ***
mean (HH income)	0.0223	0.1905 *	−0.0125	0.0528	0.0063	0.0495 ***	−0.0022	0.0087
mean (leisure)	0.1284	−0.0457	0.2200	0.3224 **	0.0362 ***	−0.0119	0.0384 ***	0.0528 ***
mean (BMI)	0.0246	0.0417 *	−0.0257	0.0250	0.0069 ***	0.0108 ***	−0.0045 ***	0.0041 ***
ln (panel variance)	−1.0673 ***	−0.9839 ***	−1.0762 ***	−1.4772 ***				
N. of individuals	6530	8693	6532	8698				
log likelihood	−3400.00	−4300.00	−2600.00	−2800.00				

Note: *** *p* < 0.001, ** *p* < 0.01, * *p* < 0.05.

**Table 4:** Estimation results: coefficient estimates of physical-related submeasures.

Variables	PF		RP		BP		GH	
	Men	Women	Men	Women	Men	Women	Men	Women
lagged dependent variable	0.7411 ***	0.4958 ***	0.3745 ***	0.3340 ***	0.4676 ***	0.4692 ***	0.4753 ***	0.3988 ***
mental (t−1)	0.2926 ***	0.2690 ***	0.4241 ***	0.2271 ***	0.3642 ***	0.2418 ***	0.3579 ***	0.2473 ***
age	−0.0164	−0.0356 **	−0.0140	−0.0225 *	−0.0350 *	−0.0364 **	−0.0604 ***	−0.0489 ***
satisfaction w. job	0.2587 ***	0.2260 ***	0.1129	0.1593 **	0.2962 ***	0.1676 **	0.3506 ***	0.2447 ***
satisfaction w. housework	0.1915 **	0.0007	0.2486 ***	0.0731	0.1079	0.0602	0.2118 **	0.1260 *
satisfaction w. HH income	0.1249	0.0369	0.0981	0.1113 *	−0.0021	0.1940 ***	0.1934 **	0.1719 **
satisfaction w. leisure	0.0729	0.1429 *	−0.0160	0.1106 *	−0.0171	0.0906	0.0577	0.2385 ***
years of education	0.0194	0.0315 ***	0.0340 **	0.0254 **	0.0534 ***	0.0405 ***	0.0213 *	0.0333 ***
white-collar	0.1311 *	0.0714	0.0756	0.1215 **	0.2065 ***	0.2091 ***	0.0370	0.0699
body mass index	−0.0692 ***	−0.0904 ***	−0.0057	−0.0391 **	0.0307	−0.0473 ***	−0.0496 *	−0.0404 **
family size	0.0170	0.0641 **	0.0176	0.0534 **	0.0412	0.0641 ***	0.0336	0.0478 *
Initial value	0.6892 ***	0.9450 ***	0.4731 ***	0.5676 ***	0.6904 ***	0.7325 ***	0.8314 ***	0.8707 ***
ln (panel variance)	−1.0905 ***	−0.8871 ***	−1.0433 ***	−1.3798 ***	−1.0215 ***	−1.3108 ***	−0.9733 ***	−0.8294 ***
N. of individuals	6532	8697	6527	8688	6534	8698	6534	8698
log likelihood	−3300.00	−4500.00	−3200.00	−4700.00	−3100.00	−4300.00	−3300.00	−4600.00

**Table 5:** Estimation results: average partial effects of physical-related submeasures.

Variables	PF		RP		BP		GH	
	Men	Women	Men	Women	Men	Women	Men	Women
lagged dependent variable	0.1810 ***	0.1320 ***	0.0858 ***	0.0890 ***	0.0967 ***	0.1091 ***	0.1201 ***	0.1060 ***
mental (t−1)	0.0766 ***	0.0733 ***	0.0954 ***	0.0625 ***	0.0780 ***	0.0602 ***	0.0922 ***	0.0670 ***
age	−0.0044 ***	−0.0099 ***	−0.0036 **	−0.0065 ***	−0.0084 ***	−0.0097 ***	−0.0163 ***	−0.0136 ***
satisfaction w. job	0.0680 ***	0.0618 ***	0.0283 ***	0.0446 ***	0.0649 ***	0.0426 ***	0.0904 ***	0.0663 ***
satisfaction w. housework	0.0508 ***	0.0002	0.0596 ***	0.0210 ***	0.0251 ***	0.0157 ***	0.0556 ***	0.0346 ***
satisfaction w. HH income	0.0334 ***	0.0102 *	0.0247 ***	0.0316 ***	−0.0005	0.0489 ***	0.0509 ***	0.0470 ***
satisfaction w. leisure	0.0196 ***	0.0393 ***	−0.0042	0.0314 ***	−0.0041	0.0235 ***	0.0155 **	0.0647 ***
years of education	0.0052 ***	0.0087 ***	0.0088 ***	0.0074 ***	0.0128 ***	0.0107 ***	0.0057 ***	0.0092 ***
white-collar	0.0350 ***	0.0197 ***	0.0192 ***	0.0344 ***	0.0466 ***	0.0525 ***	0.0099 *	0.0193 ***
body mass index	−0.0187 ***	−0.0251 ***	−0.0015	−0.0114 ***	0.0074 ***	−0.0126 ***	−0.0134 ***	−0.0112 ***
family size	0.0046 **	0.0178 ***	0.0046 **	0.0156 ***	0.0099 ***	0.0170 ***	0.0091 ***	0.0133 ***
Initial value	0.2170 ***	0.3138 ***	0.1361 ***	0.1854 ***	0.1970 ***	0.2314 ***	0.2658 ***	0.2858 ***

Note: The model additionally includes three dummies of marital status (single, divorced, and widowed), three dummies of location (west, north, and south), and correlated random effect (time average of age, job, housework, HH income, leisure, and BMI); *** *p* < 0.001, ** *p* < 0.01, * *p* < 0.05.

**Table 6:** Estimation results: coefficient estimates of mental-related submeasures.

Variables	VT		SF		RE		MH	
	Men	Women	Men	Women	Men	Women	Men	Women
lagged dependent variable	0.3516 ***	0.4257 ***	0.4103 ***	0.2879 ***	0.4944 ***	0.2421 ***	0.4638 ***	0.3335 ***
physical (t−1)	0.2549 ***	0.3294 ***	0.3547 ***	0.3584 ***	0.2715 ***	0.3306 ***	0.2473 ***	0.1678 ***
age	−0.0187	−0.0026	0.0057	−0.0235	0.0206	0.0019	0.0185	0.0089
satisfaction w. job	0.2078 *	0.2315 **	0.1913 *	0.2364 ***	0.4380 ***	0.3486 ***	0.2910 ***	0.3367 ***
satisfaction w. housework	0.1467	0.0758	0.2038 **	0.0923	0.0840	0.1150 *	−0.0289	0.0444
satisfaction w. HH income	0.0626	0.1560 *	0.2122 **	0.2458 ***	0.1048	0.2455 ***	−0.0158	0.1644 **
satisfaction w. leisure	0.1153	0.2719 ***	0.1313	0.2896 ***	0.2002 **	0.1462 **	0.2546 ***	0.4404 ***
years of education	−0.0313 **	−0.0203	0.0027	−0.0048	−0.0228 *	0.0092	−0.0005	0.0091
white-collar	−0.1905 **	−0.1750 **	0.0181	0.0751	−0.0862	0.0105	0.0090	−0.0087
body mass index	0.0353	−0.0045	0.0277	0.0018	0.0533 *	0.0077	−0.0173	−0.0070
family size	−0.0410	−0.0602 **	−0.0081	0.0305	−0.0218	0.0157	−0.0067	−0.0057
Initial value	0.6841 ***	0.6650 ***	0.6081 ***	0.7438 ***	0.5968 ***	0.6720 ***	0.4314 ***	0.5050 ***
ln (panel variance)	−0.9308 ***	−1.2695 ***	−1.0416 ***	−1.2479 ***	−1.3551 ***	−1.1997 ***	−2.0487 ***	−1.3140 ***
N. of individuals	6532	8698	6532	8696	6527	8694	6534	8697
log likelihood	−2700.00	−2900.00	−2300.00	−3400.00	−2500.00	−3900.00	−2400.00	−3400.00

**Table 7:** Estimation results: average partial effects of mental-related submeasures.

Variables	VT		SF		RE		MH	
	Men	Women	Men	Women	Men	Women	Men	Women
lagged dependent variable	0.0765 ***	0.0871 ***	0.0552 ***	0.0463 ***	0.0796 ***	0.0491 ***	0.0692 ***	0.0528 ***
physical (t−1)	0.0533 ***	0.0648 ***	0.0491 ***	0.0558 ***	0.0482 ***	0.0646 ***	0.0413 ***	0.0288 ***
age	−0.0035 ***	−0.0004	0.0009	−0.0043 ***	0.0041 ***	0.0004	0.0035 ***	0.0017 *
satisfaction w. job	0.0425 ***	0.0436 ***	0.0288 ***	0.0390 ***	0.0723 ***	0.0676 ***	0.0475 ***	0.0532 ***
satisfaction w. housework	0.0292 ***	0.0133 **	0.0305 ***	0.0163 ***	0.0162 ***	0.0245 ***	−0.0055	0.0081 *
satisfaction w. HH income	0.0120 *	0.0285 ***	0.0316 ***	0.0404 ***	0.0200 ***	0.0497 ***	−0.0030	0.0283 ***
satisfaction w. leisure	0.0227 ***	0.0522 ***	0.0204 ***	0.0466 ***	0.0367 ***	0.0308 ***	0.0424 ***	0.0661 ***
years of education	−0.0058 ***	−0.0034 ***	0.0004	−0.0009	−0.0045 ***	0.0020 **	−0.0001	0.0017 **
white-collar	−0.0324 ***	−0.0273 ***	0.0030	0.0134 ***	−0.0178 ***	0.0023	0.0017	−0.0016
body mass index	0.0066 ***	−0.0008	0.0046 ***	0.0003	0.0106 ***	0.0017	−0.0033 *	−0.0013
family size	−0.0077 ***	−0.0102 ***	−0.0013	0.0056 ***	−0.0043 **	0.0035 *	−0.0013	−0.0011
Initial value	0.1623 ***	0.1433 ***	0.1265 ***	0.1773 ***	0.1422 ***	0.1804 ***	0.0947 ***	0.1109 ***

Note: The model additionally includes three dummies of marital status (single, divorced, and widowed), three dummies of location (west, north, and south), and correlated random effect (time average of age, job, housework, HH income, leisure, and BMI); *** *p* < 0.001, ** *p* < 0.01, * *p* < 0.05.

**Table 8:** Estimation results: coefficient estimates of aggregate level in small sample.

Variables	APH				AMH			
	Men		Women		Men		Women	
	Young	Old	Young	Old	Young	Old	Young	Old
physical (t−1)	0.4652 ***	0.2160	0.3932 ***	0.5402 ***	0.2631 ***	0.3608 ***	0.3601 ***	0.3103 ***
mental (t−1)	0.3741 ***	0.5555 ***	0.3256 ***	0.3293 ***	0.4381 ***	0.2804	0.4174 ***	0.4519 **
age	−0.0346 *	−0.1117 ***	−0.0568 ***	−0.0741 **	−0.0228	−0.0013	−0.0179	0.0038
satisfaction w. job	0.2877 ***	0.5107 **	0.0682	0.5245 ***	0.1398	0.4387 **	0.1390	0.4106 **
satisfaction w. housework	0.1393	0.2126	0.1597 *	−0.1773	0.1323	0.1065	0.0857	−0.0589
satisfaction w. HH income	0.2811 ***	−0.0860	0.1044	0.3205 *	0.1365	−0.0790	0.0869	0.2490
satisfaction w. leisure	0.0533	−0.1204	0.1558 *	0.3636 **	0.1234	0.3094	0.2470 **	0.4286 **
years of education	0.0331 **	0.0151	0.0350 **	0.0205	−0.0211	−0.0367	−0.0274 *	−0.0029
white-collar	0.1602 **	0.1510	0.0920	−0.0295	−0.2339 ***	−0.1269	−0.1135	−0.1845 *
body mass index	−0.0497 *	−0.1225 **	−0.0780 ***	−0.0840 **	0.0703 **	−0.0608	−0.0171	−0.0008
family size	0.0138	0.0727	0.0402	0.0809	−0.0345	−0.1015	−0.0662 *	−0.050
Initial value	0.5951 ***	1.2101 ***	0.8185 ***	0.8522 ***	0.5329 ***	0.7546 ***	0.6027 ***	0.7214 ***
ln (panel variance)	−1.1972 ***	−0.5405	−0.9801 ***	−1.0759 *	−1.2133 ***	−0.7612	−1.5377 ***	−1.4477 *
*N*	4637	1893	5973	2720	4639	1893	5976	2722
log likelihood	−2600.00	−800	−3200.00	−1100.00	−1700.00	−790	−1800.00	−990

**Table 9:** Estimation results: average partial effects of aggregate level in small sample.

Variables	APH				AMH			
	Men		Women		Men		Women	
	Young	Old	Young	Old	Young	Old	Young	Old
physical (t−1)	0.1443 ***	0.0464 ***	0.1158 ***	0.1236 ***	0.0492 ***	0.0814 ***	0.0657 ***	0.0659 ***
mental (t−1)	0.1163 ***	0.1279 ***	0.0956 ***	0.0719 ***	0.0889 ***	0.0614 ***	0.0782 ***	0.1010 ***
age	−0.0107 ***	−0.0228 ***	−0.0162 ***	−0.0149 ***	−0.0037 ***	−0.0002	−0.0027 **	0.0007
satisfaction w. job	0.0895 ***	0.1166 ***	0.0196 *	0.1196 ***	0.0246 ***	0.1016 ***	0.0228 ***	0.0904 ***
satisfaction w. housework	0.0433 ***	0.0457 ***	0.0463 ***	−0.0339 ***	0.0232 ***	0.0218 **	0.0137 *	−0.0108
satisfaction w. HH income	0.0875 ***	−0.0172 *	0.0301 ***	0.0698 ***	0.0240 ***	−0.0150 *	0.0138 *	0.0517 ***
satisfaction w. leisure	0.0165 *	−0.0239 **	0.0452 ***	0.0800 ***	0.0215 ***	0.0685 ***	0.0427 ***	0.0950 ***
years of education	0.0102 ***	0.0031 **	0.0100 ***	0.0041 ***	−0.0035 ***	−0.0072 ***	−0.0042 ***	−0.0006
white-collar	0.0498 ***	0.0320 ***	0.0265 ***	−0.0059	−0.0337 ***	−0.0236 ***	−0.0163 ***	−0.0320 ***
body mass index	−0.0154 ***	−0.0251 ***	−0.0223 ***	−0.0169 ***	0.0115 ***	−0.0119 ***	−0.0026 *	−0.0001
family size	0.0043	0.0149 ***	0.0115 ***	0.0162 ***	−0.0056 ***	−0.0199 ***	−0.0101 ***	−0.0094 ***
Initial value	0.2026 ***	0.3299 ***	0.2670 ***	0.2123 ***	0.1076 ***	0.1889 ***	0.1165 ***	0.1729 ***

Note: The model additionally includes three dummies of marital status (single, divorced, and widowed), three dummies of location (west, north, and south), and correlated random effect (time average of age, job, housework, HH income, leisure, and BMI); *** *p* < 0.001, ** *p* < 0.01, * *p* < 0.05.

## References

[1] World Health Organization (2003). Investing in Mental Health.

[2] Osborn D.P. (2001). The Poor Physical Health of People with Mental Illness. West. J. Med..

[3] Phelan M., Stradins L., Morrison S. (2001). Physical Health of People with Severe Mental Illness. Br. Med. J..

[4] Folkins C.H., Sime W.E. (1981). Physical Fitness Training and Mental Health. Am. Psychol..

[5] Scott P.A., Schwenk T.L., Schwenk T. (2000). Physical Activity and Mental Health: Current Concepts. Sports Med..

[6] Dhaval D., Rashad I., Spasojevic J. (2008). The Effects of Retirement on Physical and Mental Health Outcomes. South. Econ. J..

[7] Wray L. (2003). Mental Health and Labor Force Exits in Older Workers: The Mediating or Moderating Roles of Physical Health and Job Factors.

[8] Evans S., Huxley P.J., Gately C., Webber M. (2006). Mental Health, Burnout and Job Satisfaction among Mental Health Social Workers in England and Wales. Br. J. Psychiatry.

[9] Fisher J.A., Sousa-Poza A. (2009). Job Satisfaction and Health: An Analysis using Panel Data and Objective Health Measures. Health Econ..

[10] Fletcher J.M., Sindelar J.L., Yamaguchi S. (2011). Cumulative Effects of Job Characteristics on Health. Health Econ..

[11] Green C.P., Heywood J.S. (2007). Performance Pay, Sorting and the Dimensions of Job Satisfaction.

[12] Godin I., Kittel F., Coppieters Y., Siegrist J. (2005). A Prospective Study of Cumulative Job Stress in Relation to Mental Health. BMC Public Health.

[13] Cai L., Kalb G. (2007). Health Status and Labour Force Status of Older Working—Age Australian Men. Aust. J. Labour Econ..

[14] Cai L. (2009). Effects of Health on Wages of Australian Men. Econ. Rec..

[15] Cai L., Cong C. (2009). Effects of Health and Chronic Diseases on Labour Force Participation of Older Working-Age Australians. Aust. Econ. Pap..

[16] Lee L.F. (1982). Health and Wage: A Simultaneous Equation Model with Multiple Discrete Indicators. Int. Econ. Rev..

[17] Stronks K., van de Mheen D., Looman C., Mackenbach J. (1996). Behavioural and Structural Factors in the Explanation of Socio-economic Inequalities in Health: An Empirical Analysis. Sociol. Health Illn..

[18] Contoyannis P., Jones A.M., Rice N. (2004). The Dynamics of Health in the British Household Panel Survey. J. Appl. Econom..

[19] Halliday T.J. (2008). Heterogeneity, State Dependence and Health. Econom. J..

[20] Ohrnberger J., Fichera E., Sutton M. (2017). The Dynamics of Physical and Mental Health in the Older Population. J. Econ. Ageing.

[21] Ohrnberger J., Fichera E., Sutton M. (2017). The Relationship between Physical and Mental Health: A Mediation Analysis. Soc. Sci. Med..

[22] Trivedi P.K., Munkin M.K., Ullah A., Giles D. (2010). Recent Developments in Cross Section and Panel Count Models. Handbook of Empirical Economics and Finance.

[23] Mundlak Y. (1978). On the Polling of Time Series and Cross Section Data. Econometrica.

[24] Wooldridge J.M. (2010). Correlated Random Effects Models with Unbalanced Panels.

[25] Cameron C., Trivedi P. (2005). Microeconometrics: Methods and Applications.

[26] Wooldridge J.M. (2005). Simple Solution to the Initial Conditions Problem in Dynamic, Nonlinear Panel Data Models with Unobserved Heterogeneity. J. Appl. Econom..

